# Control of Cyclin C Levels during Development of *Dictyostelium*

**DOI:** 10.1371/journal.pone.0010543

**Published:** 2010-05-07

**Authors:** David M. Greene, Duen-Wei Hsu, Catherine J. Pears

**Affiliations:** Department of Biochemistry, University of Oxford, Oxford, United Kingdom; University of Sevilla, Spain

## Abstract

**Background:**

Cdk8 and its partner cyclin C form part of the mediator complex which links the basal transcription machinery to regulatory proteins. The pair are required for correct regulation of a subset of genes and have been implicated in control of development in a number of organisms including the social amoeba *Dictyostelium discoideum*. When feeding, *Dictyostelium* amoebae are unicellular but upon starvation they aggregate to form a multicellular structure which develops into a fruiting body containing spores. Cells in which the gene encoding Cdk8 has been deleted fail to enter aggregates due to a failure of early gene expression.

**Principal Findings:**

We have monitored the expression levels of cyclin C protein during development and find levels decrease after the multicellular mound is formed. This decrease is triggered by extracellular cAMP that, in turn, is working in part through an increase in intracellular cAMP. The loss of cyclin C is coincident with a reduction in the association of Cdk8 with a high molecular weight complex in the nucleus. Overexpression of cyclin C and Cdk8 lead to an increased rate of early development, consistent with the levels being rate limiting.

**Conclusions:**

Overall these results show that both cyclin C and Cdk8 are regulated during development in response to extracellular signals and the levels of these proteins are important in controlling the timing of developmental processes. These findings have important implications for the role of these proteins in controlling development, suggesting that they are targets for developmental signals to regulate gene expression.

## Introduction

Cdk8 and its cyclin partner, cyclin C, are regulators of transcription through association with the mediator complex, a high molecular weight complex which couples transcriptional regulators to the basal transcription machinery [1]. Cdk8 has been postulated to have both a positive and negative role on transcription and to function either by direct phosphorylation of the C terminal domain (CTD) of RNA polymerase II or by phosphorylation of regulatory transcription factors binding to upstream promoter elements. It forms part of a sub-module of four proteins able to associate with the core mediator complex to modulate its activity. Mutation of Srb10, the *S. cerevisiae* equivalent of Cdk8, leads to altered expression of around 30% of genes suggesting this sub-module does not function at all genes but is selectively used to modulate transcription [2].

The mechanism of regulation of Cdk8 activity is not well defined, especially the role of regulation of the levels of the cyclin C subunit which is required for kinase activity. In *S. cerevisiae* proteolysis of Srb11, the orthologue of cyclin C, has been reported in response to elevated temperatures, ethanol, oxidative stress and carbon starvation [3]. The signalling pathways that result in this degradation are complex and operate upon three separate elements within the protein whose importance varies with the stimulus. These results imply that a number of independent signalling pathways act upon the Srb11 protein in response to a variety of stimuli. Alternatively, the levels of Cdk8 itself may be rate-limiting as overexpression of Cdk8 has been found to regulate β-catenin levels in colorectal cancers [4].

Cdk8 has been implicated in regulating transcription during development. In mammalian cells, cyclin C and Cdk8 are recruited to the Hairy/Enhancer of Split (HES1) developmental gene where Cdk8 hyperphosphorylates the Notch ICD (intracellular domain) – an activator of HES1 transcription. This phosphorylation results in degradation of the ICD with a resultant reduction in HES1 transcription [5]. This Cdk8-dependent proteolysis of the ICD at the promoter is analogous to the mechanism of GCN4 and Ste12 regulation by Srb10 in *S. cerevisiae* [6], [7], [8]. The concept of Cdk8 acting as a developmental gene regulator has been reinforced by a number of whole organism studies. Cdk8 is expressed in the head and trunk regions of the developing zebrafish embryo implying that it may act as transcriptional regulator in a tissue-specific manner. Similarly, the Med12 and Med13 components of the Cdk8 module have been found to be essential for correct vulva and male tail formation in *Caenorhabdtis elegans* [9] and for wing and eye morphogenesis in *Drosophila* [10], [11]. More recently, a genetic analysis in this organism indicated that, while the subunits of the Cdk8 module are not essential for cell viability, they are all necessary for the correct development of the multicellular organism [12]. The role of Cdk8 as a developmental regulator has been found to be conserved in the plant kingdom. *In Arabidopsis thaliana*, the HUA ENHANCER3 (HEN3), which encodes CdkE, a homologue of Cdk8, is involved in the proper termination of stem cells in the floral meristem and in the specification of stamen and carpel identities [13].

Cdk8 is essential to the development of *Dictyostelium discoideum* [14], [15]. This haploid organism feeds on bacteria as single cells, but upon starvation enters a multicellular life cycle [16]. Starving cells start to secrete low (nM) pulses of extracellular cAMP and surrounding cells respond to this by relaying the signal and chemotaxing up the gradient of cAMP to form a multicellular mound. This mound behaves as a multicellular organism and undergoes a number of morphogenetic events culminating in the production of a fruiting body containing a head of spores supported by a stalk of dead, vacuolated cells. *Dictyostelium* cells in which the gene encoding Cdk8 has been disrupted fail to aggregate to form mounds correlating with a failure of expression of early developmental genes [14], [15]. The requirement for Cdk8 activity early in development suggested that the activity of this protein may be developmentally regulated. Here we report that cyclin C levels are decreased during development in response to high levels of extracellular cAMP and time of development. The signalling pathway triggering the decrease in levels works through intracellular cAMP. The behaviour of *Dictyostelium* Cdk8 also alters in response to high levels of extracellular cAMP, as a decreased proportion is found associated with a high molecular weight complex. However, we see no evidence that loss of cyclin C causes the dissociation of Cdk8 but rather that cyclin C levels increase with availability of Cdk8 partner. Overexpression of cyclin C and Cdk8 was found to increase the rate of the early stages of development, consistent with the level of protein being rate-limiting.

## Materials and Methods

### Construct and strain generation

A construct to express *Dictyostelium* cyclin C with an N-terminal FLAG tag under the actin 15 promoter (pDXA[*act15::FLAG-cycC*]) was generated by amplifying the coding region of CycC from the ATG to the stop codon by PCR from genomic DNA, with the FLAG tag incorporated into the N-terminal primer. The resulting fragment was inserted into the BamHI and XhoI sites of pDXA-3C [17] to drive protein expression from the actin 15 promoter present on this extrachromosomal vector. To generate a vector to express cyclin C with an additional C-terminal TAP tag under the cyclin C promoter (pDV[*cycC::cycC-*CTAP]), this fragment was first inserted into the BglII and XbaI sites of pDV-CTAP [18]. The actin 15 promoter was then excised from this vector using SalI and BamHI and replaces with a fragment containing the 641 nucleotides upstream of the start codon of cyclin C. This fragment extends to the gene upstream of cyclin C and so is likely to contain all of the relevant control sequences for correct expression of the cyclin C gene. The construct to drive expression of *Dictyostelium* Cdk8 with an N-terminal myc tag has already been described [14].

The constructs were introduced into *Dictyostelium* Ax2 cells by electroporation and transformants selected by growth in the presence of G418 (10 µg/ml) as the expression plasmids contain the neomycin resistance gene (neoR). *GskA^-^*[*cycC::cycC-*CTAP]) and *carC^-^*[*cycC::cycC-*CTAP] cell lines were created by introducing the pDV[*cycC::cycC-*CTAP] plasmid into *gskA^-^* and *carC^-^* cells made in a Ax2 background, as previously described [19]. All strains were generated with the approval of the Biochemistry Department Genetic Modification Safety Committee, University of Oxford.

### Growth and development of *Dictyostelium*

*Dictyostelium* cells were grown axenically in HL5 medium at 22°C in shaking suspension. For development in shaking suspension, exponentially growing cells were resuspended in KK2 (16.5 mM KH_2_PO_4_, 3.8 mM K_2_HPO_4_) at 2×10^7^ cells/ml and shaken at 120 rpm and 22°C for 5 hours, pulsed with 5 nm cAMP every 5 minutes. For filter development exponential axenically growing cells were washed in KK_2_ (19 mM KH_2_PO_4_, 3.6 mM K_2_HPO_4_) and re-suspended in LPS (40 mM KH_2_PO_4_, 20 mM KCl, 680 µM dihydrostreptomycin sulphate [pH 7.2]) at 3.5×10^6^/cm^2^ on Millipore filters on an LPS soaked pad. The filters were incubated at 22°C in the dark.

### Northern and Western blot analysis

Total RNA was extracted from approximately 1×10^7^ cells using TRIZOL RNA extraction kit (Sigma) according to the manufacturer's protocol. Samples (10 µg) of total RNA were separated on a 1% formaldehyde-containing gel, blotted and probed by standard methods. Unless otherwise stated, all the northern blots are representative of at least three independent experiments. Western blot analysis was carried out as previously described [14] and blots shown are representative of at least three independent experiments.

### Size fractionation

1×10^9^ cells were harvested washed twice with KK_2_ and lysed in 4 ml of nuclear isolation (NI) buffer (50 mM Tris-HCl [pH 7.5], 5 mM magnesium acetate, 10% Sucrose, 2% NP-40 and protease inhibitor cocktail (Roche)) and centrifuged at 2,300×g in order to collect the nuclei. The nuclei were resuspended in 250 µl of NI buffer before addition of an equal volume of (10 mM HEPES [pH 7.9], 600 mM NaCl, 10% sucrose, 5 mM MgCl_2_, 0.1 mM EDTA, 0.5% NP-40, 1 mM DTT and protease inhibitor cocktail). After spinning to clear debris, the supernatant was subjected to gel filtration on a 24 ml Superose 6 column (Amersham Pharmacia Biotech) that had been equilibrated in gel filtration buffer (25 mM HEPES [pH 7.4], 300 mM NaCl, 1 mM EDTA, 10 mM β-mercaptoethanol, 5% glycerol). Fractions of 1 ml were collected and analysed by western blot. The size of protein complexes in each fraction was calculated by using the High Molecular weight gel filtration calibration kit (GE Healthcare).

## Results

### Identification of *Dictyostelium* Cyclin C

**Figure 1:**
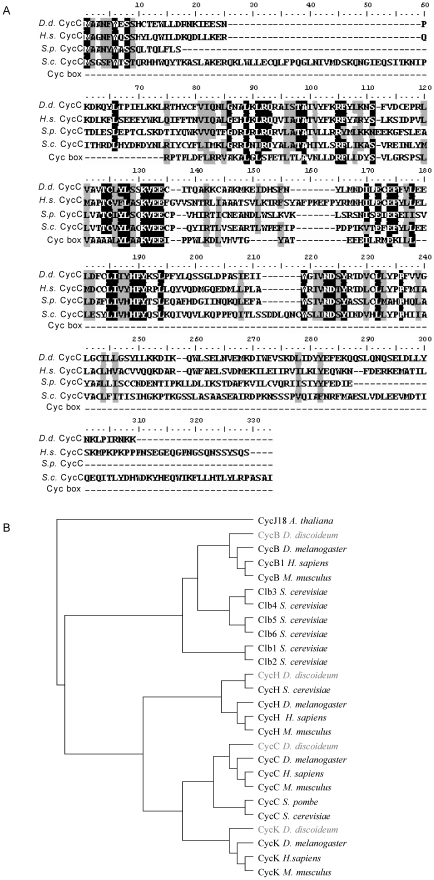
Comparison of cyclin C coding sequences. **A**. The sequence of the predicted *Dictyostelium* protein (DDB_G0274139 at dictybase.org) was aligned against *Homo sapiens* (*H.s*.) Cyclin C and Srb11 from *S. pombe* (*S.p*.) and *S. cerevisiae* (S.c.). Alignment against a generic cyclin box from the NCBI conserved domain database (cd00043) is also shown. **B**. Cyclin protein sequences were obtained from NCBI (www.ncbi.nlm.nih.gov) nucleotide databases and aligned using Clustal W in Bioedit [36]. A generic cyclin box sequence from the NCBI conserved domain database (cd00043) was used to define the cyclin box domain within each protein. These domains were used to construct a phylogenetic tree in Bioedit using the neighbour joining method. The tree was rooted using CycJ18 from *Arabidopsis thaliana* as an outgroup.

A putative cyclin C gene (*cycC*) was identified by a BLAST search of the *Dictyostelium* genomic database (Dictybase ID: DDB_G0274139 at dictybase.org). The protein contains the characteristic α-helical cyclin box fold domain and exhibits 45% amino acid identity and 62% similarity to the human cyclin C protein (Figure 1A). These levels of similarity were higher than when compared with the *S. pombe* protein (33% identity, 51% similarity) or the *S. cerevisiae* protein (32% identity, 46% similarity). In order to identify the evolutionary history of the putative *Dictyostelium* CycC protein, the cyclin box domains present in a number of cyclin proteins was used to plot a phylogenetic tree (Figure 1B). The *Arabidopsis thaliana* cyclin CycJ18 was used as an out group to root the tree. Cyclin C homologues that had been identified in other organisms were placed in the same clade as the *Dictyostelium* CycC protein indicating that they shared a common ancestor.

### Expression of *Dictyostelium* cyclin C

**Figure 2:**
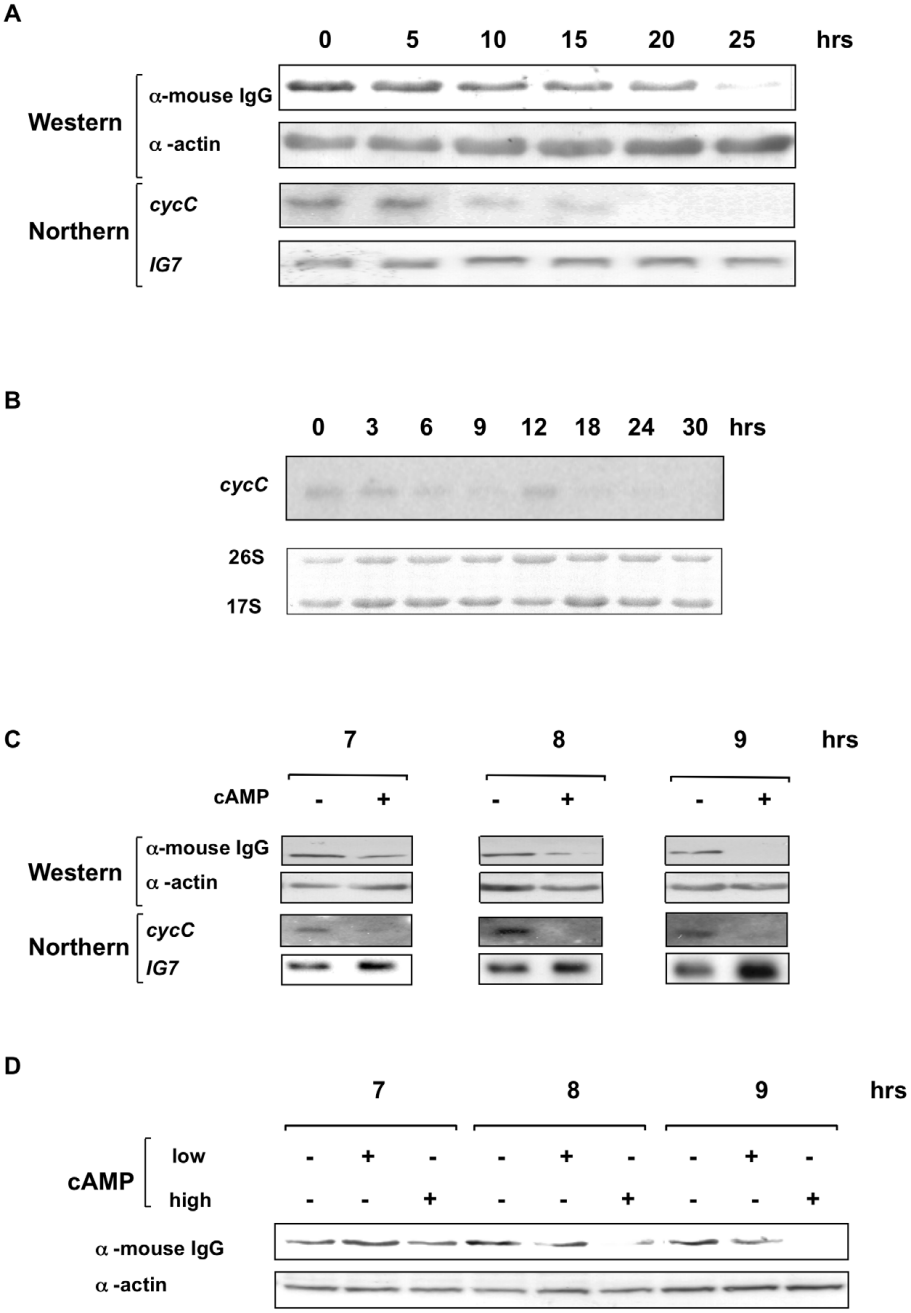
Developmental expression of epitope-tagged cyclin C from its own promoter. **A**. Exponentially growing cells overexpressing tagged cyclin C from its own promoter (Ax2[*cycC*::*cycC*-CTAP] cells) were harvested, washed and developed on filters at a density of 3×10^6^ cells/cm^2^ and samples harvested for western and northern blots at the times indicated. Development was complete after 24 hours. Western samples were analysed on a 10% SDS-PAGE gel, transferred to a nitrocellulose membrane and probed with an α-mouse IgG secondary antibody (as the TAP tag present in the protein contains an IgG binding domain) and an α-actin antibody to control for loading. RNA samples were resolved on a 1% formaldehyde gel, transferred to a nylon membrane and probed with a ^32^P labelled fragment of the *cycC* gene and the *IG7* gene (to control for loading). The blot was exposed overnight to reveal cycC expression. B. Exponentially growing Ax2 cells were harvested and developed on filters as described above. RNA was isolated at the times shown and probed with a fragment of the cycC gene, The blot was exposed for 10 days. The levels of 17S and 26S rRNA are shown as a control for equal loading. **C**. Ax2[*cycC*::*cycC-*CTAP] cells were shaken in KK_2_ buffer at 5×10^6^ cells/ml and pulsed with 50 nM cAMP every 5 mins. After 4 hrs cells were split and incubated in the presence (+) or absence (−) of 500 µM cAMP (added every hr for the duration of the experiment). Samples were taken at the time points indicated and analysed by western and northern blot as in Figure 1. **D**. Ax2[*cycC*::*cycC-*CTAP] cells were shaken in KK_2_ buffer and pulsed with 50 nM cAMP as above. After 4 hrs the cells were split into three. One sample acted as an untreated control, one was pulsed with 50 nM cAMP as before (low cAMP), and the third treated with 500 µM cAMP (high cAMP) every hour for the duration of the experiment. Samples were analysed by western blot as described above.

The pDV[*cycC::cycC*-CTAP] plasmid was constructed to express the *Dictyostelium* cyclin C protein with a C-terminal tandem affinity purification tag (CycC-CTAP) from its endogenous *cycC* promoter. This promoter fragment stretches 641 nucleotides upstream of the *cycC* coding sequence to the terminus of the neighbouring gene, and is therefore likely to contain all the elements required for correctly regulated *cycC* expression. This pDV[*cycC::cycC*-CTAP] plasmid was transformed into *Dictyostelium* Ax2 cells and transformants selected by neomycin resistance to generate Ax2[*cycC*::*cycC-*CTAP] cells. Western blot of a whole cell extract from this strain was able to detect a protein of the predicted size of CycC-TAP. The Ax2[*cycC*::*cycC*-CTAP] cells were used to assess the levels of cyclin C expression throughout the developmental life cycle by western blot using antisera directed against the tag (Figure 2A). The protein was expressed in growing cells and the level of tagged cyclin C protein declined during later development starting at around the stage of tipped aggregate formation. This loss of protein was matched by a loss in mRNA levels consistent with the regulation of protein levels being due, at least in part, to loss of mRNA (Figure 2A). This analysis will detect the transcripts from both the endogenous cyclin C gene and that expressed from the plasmid. However, at the exposure times used to detect expression of the transgene, no endogenous cyclin C mRNA expression was detectable (data not shown). Longer exposure of a developmental time course northern of parental Ax2 cells revealed that the endogenous cyclin C mRNA showed a similar pattern of expression to the transgene, with reduced levels later in development, consistent with the transgene having all the regulatory sequences required for correct expression (Figure 2B). All further northern blots shown are short exposures and are likely, therefore, only to be detecting transgene expression, although it is possible the endogenous transcript contributes to the signal.

The level of extracellular cAMP is known to rise to mM concentration in the mound at around the time the loss of cyclin C is observed. In order to assess whether this increase in cAMP levels is the trigger for loss of cyclin C, cells were developed in shaking suspension in the presence of low (nM) cAMP pulses to mimic the early stages of development. In these cells the loss of tagged cyclin C protein and mRNA can be induced by addition of high concentrations of extracellular cAMP similar to those experienced in the mound (Figure 2C). Longer pulsing with lower levels of cAMP does not induce loss of cyclin C expression (Figure 2D) and high cAMP levels do not accelerate loss of cyclin C at earlier stages of development (data not shown). These data, therefore, suggest that during the earlier stages of development cells become competent to respond to high levels of extracellular cAMP, as experienced in the mound, in order to reduce levels of cyclin C.

### Developmental regulation of *Dictyostelium* cyclin C levels by intracellular cAMP

**Figure 3:**
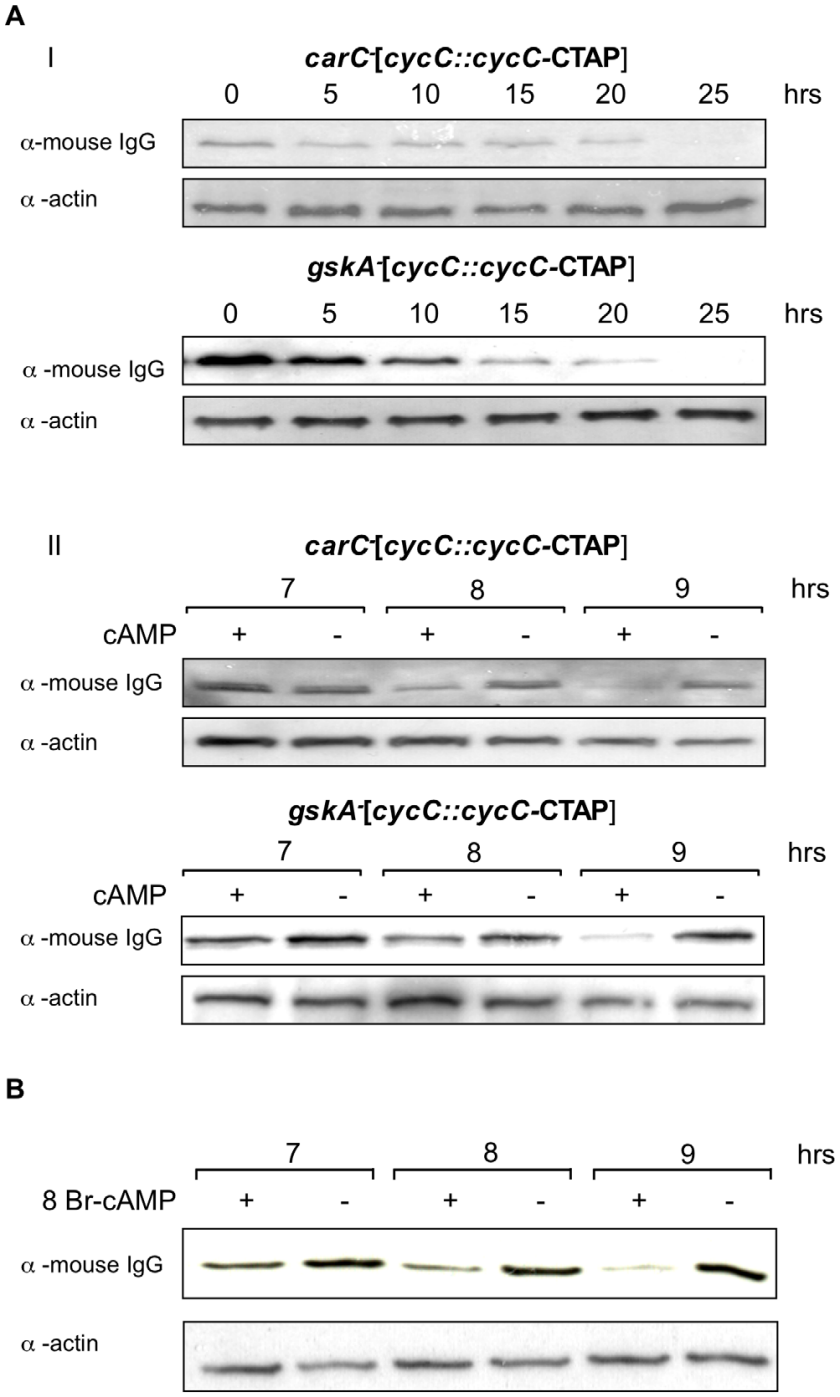
Signals regulating expression of cyclin C during development. **A**. **I**. Exponentially growing *carC^-^*[*cycC::cycC-*CTAP] and *gskA^-^*[*cycC::cycC-*CTAP] cells were harvested and developed on filters at a density of 3×10^6^ cells/cm^2^. Samples were collected at the times indicated and analysed by western blot as in Figure 1A. **II**. Exponentially growing *carC^-^*[*cycC::cycC-*CTAP] and *gskA^-^*[*cycC::cycC-*CTAP] cells were harvested and shaken in KK_2_ buffer at 5×10^6^ cells/ml and pulsed with 50 nM cAMP every 5 mins. After 4 hrs cells were split and shaken in the presence (+) or absence (−) of 500 µM cAMP, added every hour for the duration of the experiment. Samples were taken at the time points indicated and analysed by western blot as above. **B**. Exponentially growing Ax2[*cycC*::*cycC-*CTAP] cells were harvested and shaken in KK_2_ buffer at 5×10^6^ cells/ml, pulsed with 50 nM cAMP every 5 mins. After 4 hrs the cells were split and incubated in the presence (+) or absence (−) of 5 mM 8Br-cAMP. Samples were collected at the times indicated and analysed by western blot as in Figure 1A.

Two signalling pathways have been implicated downstream of extracellular cAMP at the stage of development at which cyclin C levels decline, both working through seven transmembrane domain cAMP receptors: activation of the serine/threonine kinase GskA (the *Dictyostelium* orthologue of GSK3) and, through increase in intracellular cAMP, activation of cAMP-dependent protein kinase [20], [21]. We set out to determine if either of these pathways was responsible for the loss in cyclin C observed. GskA is activated by tyrosine phosphorylation by zak1 downstream of the extracellular cAMP receptor cAR3, one of a number of cAMP receptors expressed at this stage of development. Therefore we expressed tagged cyclin C, driven by its endogenous promoter, in *gskA^-^* (*gskA^-^*[*cycC::cycC-*CTAP]) and *carC^-^* (*carC^-^*[*cycC::cycC-*CTAP]) cells to see if levels of cyclin C could still be reduced by treatment with high extracellular cAMP. In cells lacking the genes encoding GskA or cAR3, the loss of cyclin C in response to high extracellular cAMP was seen as in parental Ax2 cells, suggesting that this pathway is not essential for the reduction in levels (Figure 3A), although we cannot rule out that it may alter the efficiency. In order to investigate the role for intracellular cAMP, Ax2[*cycC*::*cycC*-CTAP] cells developed in shaking suspension were exposed to 8Br-cAMP. This membrane permeable cAMP analogue does not activate the extracellular cAMP receptors but enters the cells and directly activates cAMP-dependent protein kinase [22]. Addition of 8Br-cAMP to cells at the relevant stage of development is sufficient to bring about a decrease in cyclin C levels, and, as previously, there is a simultaneous decrease in the levels of both cyclin C mRNA and protein (Figure 3B). This implicates intracellular cAMP levels in the pathway leading to down-regulation of cyclin C expression in mid-development, although this may not be the only important factor and the rate and extent of loss may be dependent on other pathways.

### *Dictyostelium* cyclin C levels do not alter in response to oxidative stress

**Figure 4:**
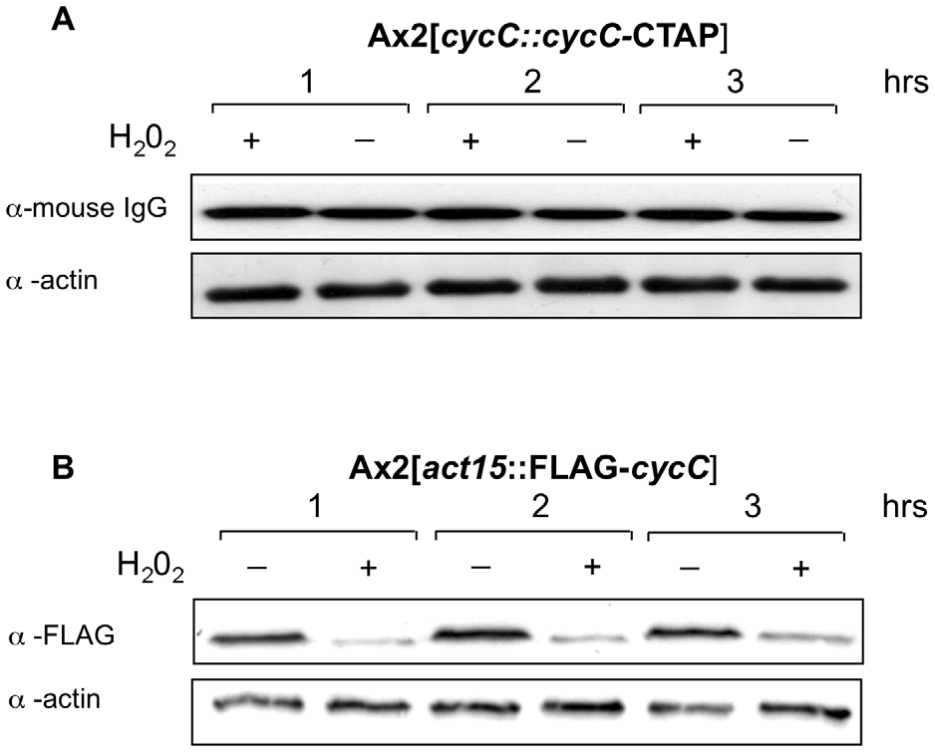
CycC-CTAP protein levels in response H_2_O_2_ treatment. Ax2[*cycC*::*cycC*-CTAP] and *Ax2*[*act15::*FLAG*-cycC*] cells were harvested, washed and shaken in KK_2_ buffer at 1×10^7^ cells/ml. After 1 hr the cells were split and incubated in the presence (+) or absence (−) of 0.25 mM H_2_O_2_. Western samples were taken after 1, 2 and 3 hrs of treatment and analysed on a 10% SDS-PAGE gel, transferred to a nitrocellulose membrane and probed with an α-mouse IgG secondary antibody and an α-actin antibody to control for loading.

In *S. cerevisiae*, cyclin C levels have been reported to respond to a number of cellular stresses such as oxidative stress although this has not been reported in other systems. In order to determine if this is a universal phenomenon, we investigated the levels of epitope-tagged cyclin C in cells following treatment with hydrogen peroxide at a concentration known to induce approximately 25% cell death [23]. When expressed from its endogenous promoter the levels of cyclin C protein and mRNA were unaltered following H_2_O_2_ treatment (Figure 4 and data not shown). When the cyclin C was expressed from the actin 15 promoter, however, the level of protein did decrease following H_2_O_2_ treatment. This was mirrored by a decrease in mRNA levels (data not shown), suggesting that the oxidative shock was leading to a decrease in mRNA levels which led to a subsequent decrease in protein levels. The down-regulation of the actin 15 promoter confirms that the cells were responding to oxidative stress but the fact that cyclin C mRNA and protein levels remained unchanged when expressed from the endogenous cyclin C promoter indicates that the pathway inducing loss of cyclin C in response to these levels of oxidative stress is not present in *Dictyostelium*.

### Association of *Dictyostelium* cyclin C and Cdk8 with high molecular weight complexes

**Figure 5:**
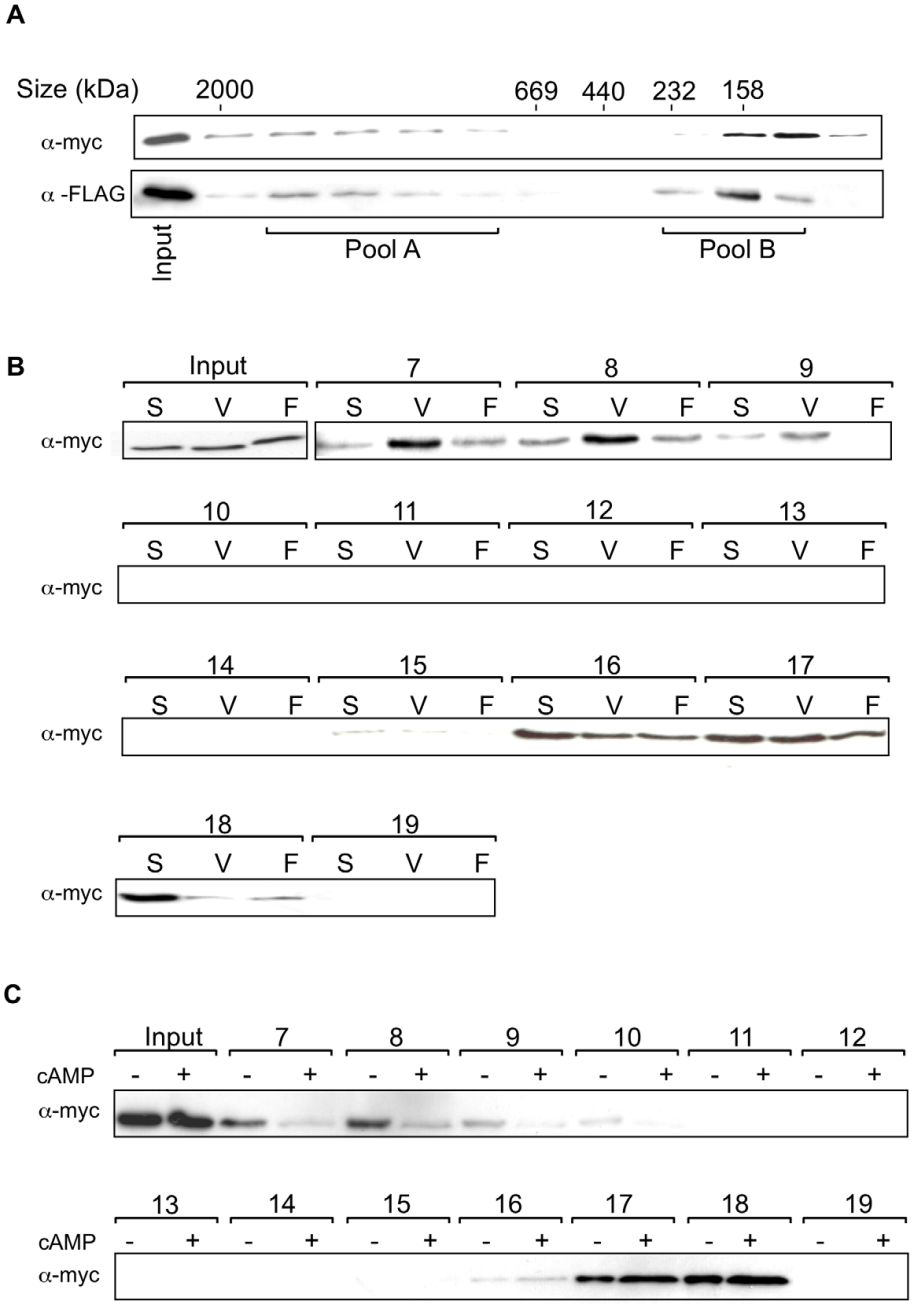
Size fractionation of complexes containing FLAG-CycC and myc-Cdk8. **A**. 1×10^9^ vegetatively growing Ax2[*myc-cdk8*, FLAG-*cycC*] cells were harvested and the extracted nuclei lysed and subjected to gel filtration on a Superose 6 column. Fractions (7–18) were resolved on a 12% SDS-PAGE gel, transferred to a nitrocellulose membrane and probed with α-FLAG and α-myc antibodies. Positions of molecular weight standards are specified above. **B**. 1×10^9^ Ax2[*myc-cdk8*] cells were harvested after either vegetative growth (V), development in shaking suspension for 8 hours with high (500 µM) cAMP added after 4 hours, as in Figure 2, (S) or development on filters up to the first finger stage (F). A nuclear extract from each of these cells was fractionated by gel filtration on a Superose 6 column. Samples of fractions 7 to 19, as indicated, were resolved on a 12% SDS-PAGE gel, transferred to a nitrocellulose membrane and probed with an α-myc antibody. **C**. 2×10^9^ Ax2[*myc-cdk8*] cells were resuspended in KK_2_ buffer and pulsed with 50 nM cAMP for 4 hrs. Cells were then split and either left untreated (−) or treated with 500 µM cAMP every hour (+) until the cells were harvested at 8 hrs. Nuclei were isolated, lysed and analysed by gel filtration chromatography and western blot as above.

Cyclin C and its kinase partner, Cdk8, form part of the high molecular weight mediator complex in mammalian cells and yeast. In order to determine if the *Dictyostelium* proteins are also found in a complex, a construct to drive expression of *Dictyostelium* Cdk8, tagged with an epitope from c-myc at its N terminus, was transfected into Ax2 cells either on its own or co-transfected with a construct to drive expression of tagged cyclin C. Therefore cells were generated overexpressing cyclin C, Cdk8 or both. Gel filtration analysis of nuclear extracts from cells expressing both epitope-tagged Cdk8 and cyclin C revealed a high proportion of these proteins as part of a high molecular weight complex (Figure 5A). Protein also elutes in fractions consistent with a lower molecular weight which might represent free subunit or protein only complexed to a smaller number of interacting proteins. In mammalian cells, only a very small proportion of Cdk8 is found outside the high molecular weight complex [24], so this lower molecular weight fraction could be a consequence of overexpressing the protein, saturating the availability of interacting partners. In order to determine if the reduction in cyclin C levels during development was associated with a reduction in the proportion of Cdk8 found in the high molecular weight complex, the fraction of Cdk8 eluting at high molecular weight was compared in growing cells with cells from two conditions where a reduction in cyclin C levels is seen; firstly following development in shaking suspension and treatment with high extracellular cAMP and secondly following multicellular development to the first finger stage (Figure 5B). The first finger stage is around 15–18 hours of development at a time when the levels of cyclin C are seen to be reduced compared to growing cells (Figure 2A). It is not feasible to carry out the experiment in the mature fruiting body when cyclin C levels were further reduced, as it is difficult to lyse the differentiated stalk and spore cells in conditions that would leave protein complexes intact. In both conditions where reduced levels of cyclin C are apparent, a lower proportion of Cdk8 is found in the high molecular weight complex, demonstrating an alteration in Cdk8 behaviour as cyclin C levels decrease. Comparison of cells in shaken suspension in the presence and absence of high extracellular cAMP demonstrates that this signal, at the relevant stage of development, is sufficient to reduce the association of Cdk8 (Figure 5C).

**Figure 6:**
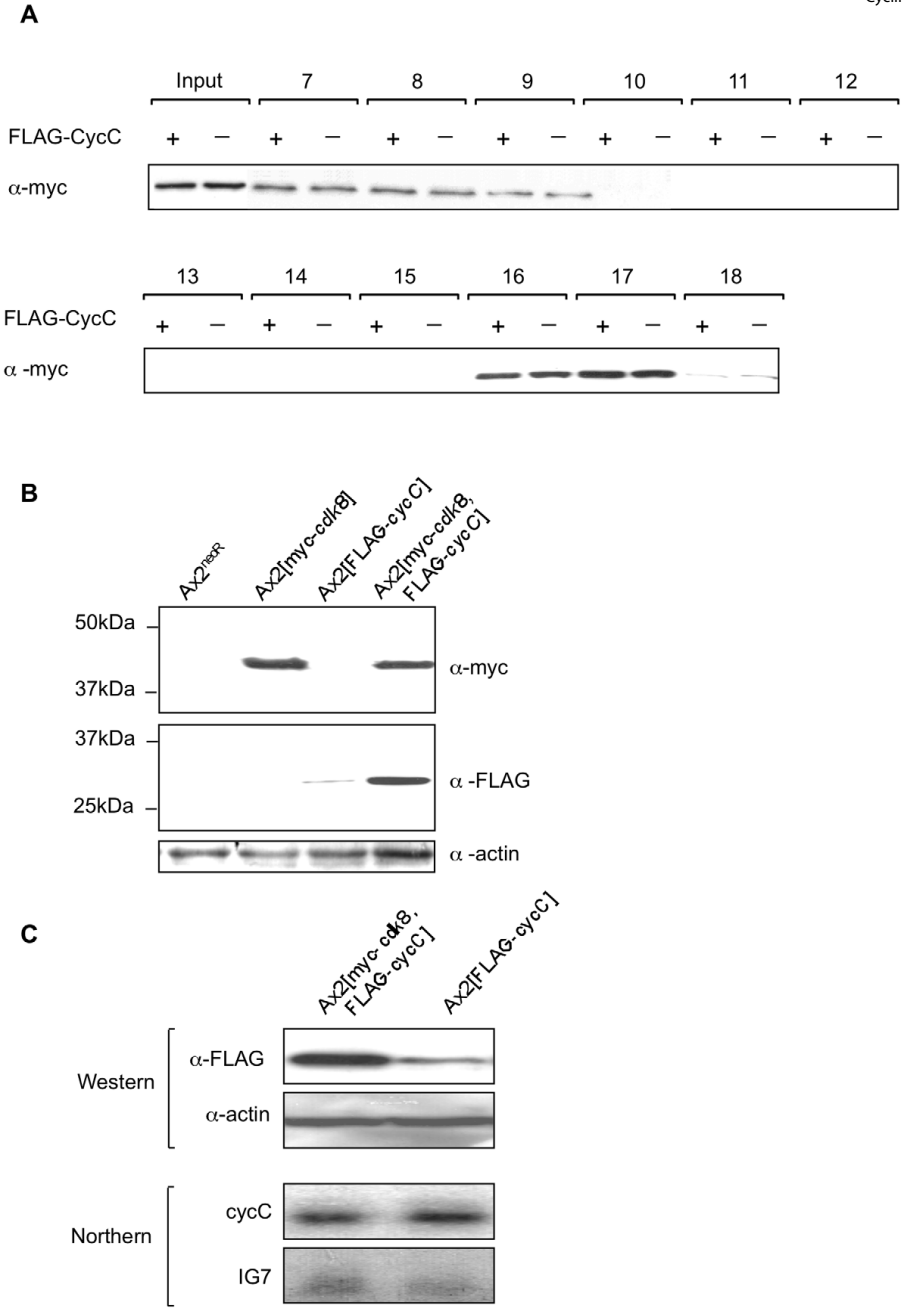
Analysis of myc-Cdk8 complexes in the presence and absence of FLAG-CycC. **A**. Nuclear extracts from 1×10^9^ Ax2[*myc-cdk8*, FLAG*-cycC*] or Ax2[*myc-cdk8*] cells (annotated as FLAG-CycC + and - respectively) were fractionated by gel filtration on a Superose 6 column and analysed as described in the legend to Figure 5. **B and C**. Samples were harvested from vegetatively growing cells and analysed by western (B and C) and northern (C) blot. Western blots samples were analysed and probed with the α-FLAG antibody to recognise FLAG-CycC and against the myc epitope to recognise myc-Cdk8. RNA samples were resolved on a 1% formaldehyde gel, transferred to a nylon membrane and probed with a ^32^P labelled fragment of the *cycC* gene. The blot was reprobed with a ^32^P labelled fragment of the *IG7* gene to control for loading.

**Figure 7:**
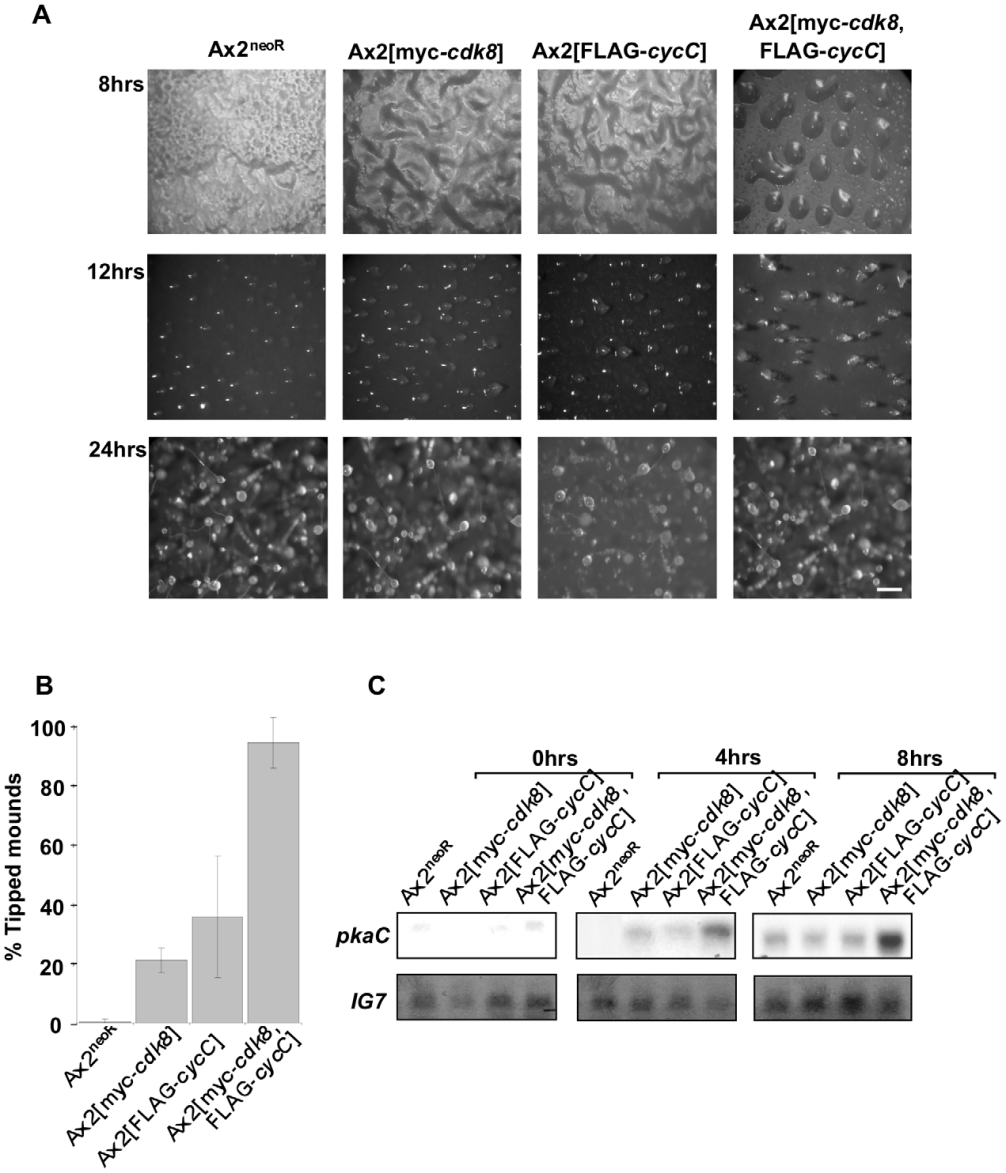
Developmental phenotype of Cdk8-myc and CycC-FLAG expressing strains. **A**. Cells of strains Ax2[*myc-cdk8*, FLAG*-cycC*], Ax2[*myc-cdk8*], *Ax2*[FLAG*-cycC*] and Ax2 cells containing an empty vector control (Ax2^neoR^) were harvested from exponential growth, developed on filters at a density of 3×10^6^ cells/cm^2^ and photographed at the times indicated. The scale bar in the bottom right panel represents 300 µm. **B**. To quantify differences in developmental rate, aggregates were scored for the presence or absence of a tip after 13 hrs of development. Each bar represents the mean (±standard deviation) of 3 independent experiments, in which a minimum of 200 aggregates were scored for each strain. **C**. Cells were harvested from filters at the times indicated and RNA extracted and analysed by Northern blot. RNA samples were resolved on a 1% formaldehyde gel, transferred to a nylon membrane and probed with a ^32^P labelled fragment of the *pkaC* gene. The blot was reprobed with a ^32^P labelled fragment of the *IG7* gene to control for loading. This northern is representative of two independent experiments.

One explanation for this data is that the level of cyclin C is the determining factor for Cdk8 association with the complex, so as cyclin C levels decline following transcriptional down-regulation, an increasing proportion of Cdk8 dissociates. To test this we compared the proportion of tagged Cdk8 eluting in the high molecular weight fraction in cells with normal levels of endogenous cyclin C and cells overexpressing epitope-tagged cyclin C from the strong, constitutive actin 15 promoter (Figure 6A). There was no difference in the level of Cdk8 found in the high molecular weight fraction suggesting that the association of Cdk8 with the complex is not purely determined by the level of cyclin C present in the cell. Conversely, co-expression of tagged cyclin C and Cdk8 in the same cells led to a higher level of stable cyclin C protein. When the levels of cyclin C mRNA expressed from the transgene were compared in cells overexpressing cyclin C alone with that in cells overexpressing both cyclin C and Cdk8 the mRNA levels were equivalent in the presence or absence of Cdk8 overexpression. However, western analysis revealed a higher level of cyclin C protein in cells which also overexpressed Cdk8 indicative of a post-transcriptional effect of Cdk8 levels on cyclin C protein levels (Figure 6B). This is consistent with Cdk8 leading to stabilisation of the cyclin C protein. This increase in levels has consequences for development as the rate of early development is considerably accelerated in overexpressing cells (Figure 7A,B). Overexpression of either cyclin C or Cdk8 individually leads to an acceleration of early development but overexpression of both decreases the time at which aggregates form still further such that the majority (94%) of aggregates had developed a tip by 13 hours of development in the double overexpressing strain compared to 0.5% in the control strain. This accelerated development is confirmed by earlier expression of a gene up-regulated during early development, *pkaC* (Figure 7C).

## Discussion

Since its identification as a component of the RNA polymerase II holoenzyme, Cdk8 has been implicated as both a positive and negative regulator of transcription. Much of this analysis has been conducted in yeast and has demonstrated that Cdk8 is not a global regulator of gene expression but is instead involved in regulating gene subsets involved in meiosis and response to environmental stresses [25], [26]. Increasingly, Cdk8 has been implicated in the regulation of metazoan development. Studies have detected localised expression of Cdk8 in zebrafish embryos [27], involvement of Cdk8 in the Notch cell fate signalling pathway [5] and a requirement for Cdk8 during Drosophila leg and eye development [12]. These studies are consistent with analysis in *Dictyostelium* which demonstrated that Cdk8 deficient strains exhibit defects in the early aggregation stages of development [14], [15].

In a number of studies, across a range of organisms, the regulatory partner of Cdk8 has been shown to be Cyclin C [28], [29], [30]. A bioinformatics approach was used to identify a *Dictyostelium* orthologue of this protein which contained the cyclin box fold domain and exhibited high levels of identity and similarity to both human and yeast cyclin C proteins. Upon phylogenetic analysis, the putative *Dictyostelium* cyclin C protein fell into the same clade as cyclin Cs from other organisms suggesting that it shares a more recent common ancestor with these proteins than with other cyclins.

As cyclins are typically regulated by controlling their abundance within the cell, a study was undertaken to determine whether cyclin C was regulated by this mechanism. To achieve this, it was necessary to develop a system to detect the protein when expressed from its endogenous *cycC* promoter. Detection of the endogenous cyclin C protein was attempted by generating a strain in which an epitope tag was inserted into the endogenous gene. However, although Southern analysis of this strain and RT-PCR analysis were consistent with successful insertion and expression of the resulting mRNA, it was not possible to detect protein by western blot using antisera against the epitope tag, even after attempts to enrich by immunoprecipitation (unpublished observations). This is consistent with the reports of low abundance of the mediator complex [31]. In order to obtain detectable levels of protein it was necessary to overexpress tagged protein from its endogenous promoter, including all the sequence upstream of the cyclin C coding sequence as far as the next gene. Such regions have been shown to contain all the necessary sequences for correct developmental regulation of expression of a number of developmentally regulated genes in *Dictyostelium* and there have been no reports of other regions being required. Overexpression was achieved by the presence of multiple copies of the plasmid in transformed cells, as a result of selection for neomycin resistance by G418 in *Dictyostelium* [32]. Overexpression of mRNA was confirmed by Northern analysis as the endogenous transcript was not detected at exposures at which the transgene gave a robust signal. However, on long exposure of developmental time course of Ax2 cells, the endogenous cyclin C mRNA levels showed a similar pattern of decreased expression during development.

As Cdk8 has been implicated in *Dictyostelium* development, it was hypothesised that cyclin C levels may be altered during this process to control Cdk8 activity. In support of this, CycC-CTAP protein levels were observed to decrease throughout development and further analysis suggested that the decrease was promoted by high levels of extracellular cAMP which mimics conditions found at the mound stage of the life cycle. As *Dictyostelium* development is a consequence of starvation it could be argued that the reduction in CycC-CTAP levels was a reaction to nutrient depletion similar to that observed in yeast [3]. However, the reduction in CycC-CTAP abundance occurred in response to the developmental signalling molecule cAMP suggesting that it is a genuine developmental regulatory event. Northern analysis revealed that expression of the *cycC*-CTAP transcript decreased throughout development and in response to high extracellular cAMP. This suggested that CycC-CTAP levels were regulated at least in part by changes in mRNA expression rather than protein stability although additional regulation at this level cannot be excluded. However, when expressed from either its endogenous promoter or that of the actin 15 gene, we always observed cyclin C protein levels to mimic the abundance of the mRNA during both development and in response to stress (Figures 2,3,4 and unpublished observations). This would be consistent with reports in human cells that cyclin C protein has a short half life and cyclin C levels may be regulated by changes in gene expression rather than protein stability [33], [34]. Unlike in *S. cerevisiae*, we could not find any change in cyclin C levels in response to oxidative stress in *Dictyostelium* under the experimental conditions used.

Size fractionation of myc-Cdk8 and FLAG-CycC complexes indicated that the two proteins were both present in a complex of between 669 KDa and 2 MDa in size, which is similar to the reported size of the many Cdk8 and cyclin C containing mediator complexes that have been isolated from other organisms. During later development and in response to high extracellular cAMP, the proportion of Cdk8 associated with the high molecular weight complex decreased. Although these events correlated with the decrease in CycC-CTAP levels, additional analysis hinted that cyclin C abundance may not be the only factor which controls association of Cdk8 with the large protein complex: When a cyclin C protein was overexpressed in the same cells as overexpressed myc-Cdk8 it was found that there was no increase in the level of myc-Cdk8 in the large complex. While this may be because the mediator complex is already saturated with myc-Cdk8, it might also suggest that association of Cdk8 with the complex is regulated by additional signalling pathways and that the level of cyclin C is not rate limiting.

In human cells it has been demonstrated that, unlike other cyclins, cyclin C is stabilised by an interaction with the Cdk8 protein [35]. Consequently, it was predicted that if the *Dictyostelium* Cdk8 and CycC proteins are functional partners then the levels of cyclin C would be higher in cells which also expressed the myc-Cdk8 protein. Western blotting indicated that this was the case, while northern analysis confirmed that this was not due to transcriptional regulation of the *act15* promoter from which both proteins were expressed. Increasing the levels of either cyclin C or Cdk8 individually was sufficient to increase the rate of early development, though overexpression of both led to a greater acceleration. This is consistent with the levels of the cyclin C and Cdk8 being rate limiting for early development, although the proportion of Cdk8 found in the high molecular weight complex did not change on co-expression of cyclin C.

In conclusion we report a decrease in cyclin C levels in response to an extracellular developmental signal working through a cell surface receptor. These experiments demonstrate the importance of Cdk8 and cyclin C in controlling developmental processes, and that the levels of Cdk8 are rate-limiting for early development. Understanding the signalling events controlling the levels of these proteins and their association with other complex members is an important aspect of regulation of transcriptional events during development and differentiation.
